# Time Will Show: Real Time Predictions during Interpersonal Action Perception

**DOI:** 10.1371/journal.pone.0054949

**Published:** 2013-01-22

**Authors:** Valeria Manera, Ben Schouten, Karl Verfaillie, Cristina Becchio

**Affiliations:** 1 Center for Cognitive Science, Department of Psychology, University of Turin, Turin, Italy; 2 Laboratory of Experimental Psychology, K.U. Leuven, Leuven, Belgium; Royal Holloway, University of London, United Kingdom

## Abstract

Predictive processes are crucial not only for interpreting the actions of individual agents, but also to predict how, in the context of a social interaction between two agents, the actions of one agent relate to the actions of a second agent. In the present study we investigated whether, in the context of a communicative interaction between two agents, observers can use the actions of one agent to predict when the action of a second agent will take place. Participants observed point-light displays of two agents (A and B) performing separate actions. In the communicative condition, the action performed by agent B responded to a communicative gesture performed by agent A. In the individual condition, agent A's communicative action was substituted with a non-communicative action. For each condition, we manipulated the temporal coupling of the actions of the two agents, by varying the onset of agent A's action. Using a simultaneous masking detection task, we demonstrated that the timing manipulation had a critical effect on the communicative condition, with the visual discrimination of agent B increasing linearly while approaching the original interaction timing. No effect of the timing manipulation was found for the individual condition. Our finding complements and extends previous evidence for interpersonal predictive coding, suggesting that the communicative gestures of one agent can serve not only to predict *what* the second agent will do, but also *when* his/her action will take place.

## Introduction

Perception of the actions of conspecifics works by prediction. At the most basic level, from seeing the start of a movement, human observers can anticipate how it will end [1]. For instance, by looking at a person throwing a dart on a target board, observers can predict the landing position of the dart on the board [2]. Similarly, observers can anticipate the direction and depth of a badminton or a tennis stroke [3]–[7], predict the fate of a basketball shot [8] or determine whether a player is about to throw a ball or mimic a throw [9]. In more complex situations, predictive processes allow humans to understand others' intentions and anticipate what they will do next [10]. For example, from seeing someone grasping an object, observers can anticipate whether the object is grasped with the intent to cooperate, compete, or perform an individual action [11], [12].

Recent evidence suggests that action anticipation is crucial not only for interpreting the actions of individual agents, but also to predict how, in the context of a social interaction between two agents, the actions of one agent relate to the actions of a second agent. This was first demonstrated by Neri, Luu, and Levi [13] in the context of interpersonal activities requiring close-body contact such as fighting or dancing. Participants observed point-light displays of two fighters masked with noise dots scattered all over the screen. Visual detection of the target agent was better when the agent was embedded in a fighting sequence with the second agent acting synchronously as opposed to asynchronously, even though synchronization was irrelevant to the visual discrimination task. Manera and colleagues [14], [15] reported a similar effect for communicative interactions, in which no physical contingency is implied between the movements of two interacting agents. Observing the communicative gesture of the first agent enhanced visual detection of the second agent in a simultaneous masking detection task. These findings suggest that in the context of a social interaction the actions of one agent serve as predictors for the expected actions of the other agent [14]. However, little is known about the *timing* of this ‘interpersonal predictive coding’ during the observation of communicative interactions. Do observers use the communicative actions of one agent to predict *when* the action of the second agent takes place?

It has been proposed that inferences about the course of actions are based on real-time predictions, running time-locked to the observed action [16]. In accordance with this proposal, prediction of temporally occluded actions has been shown to be best for actions in which the occluder duration matches the movement gap, i.e., the duration of the unseen part of the action [17], [18]. The aim of the present study was to investigate whether real time action prediction mechanisms extend to interacting dyads.

Time poses serious constraints on many interactive activities. Indeed, as the time window for coordination is often very narrow, actors must achieve a close temporal coordination for acting synchronously or in turn [19], [20]. We reasoned that if interpersonal predictive coding is sensitive to these timing aspects of interactions, then altering the timing of the action of one agent should affect the processing of the action of the other agent. To evaluate this hypothesis, we systematically manipulated the timing of the actions of two agents in a simultaneous masking detection task.

Participants observed point-light displays of two agents (A and B) performing separate actions. In the communicative condition, the action performed by agent B (squatting down) responded to a communicative gesture performed by agent A (asking to squat down). In the individual (control) condition, agent A's communicative action was substituted with a non-communicative, unrelated action (turning). For each type of action sequence (communicative vs. individual), we varied the onset of agent A's action, in order to obtain three different time sets (‘+0’, ‘+20’, ‘+40’). If interpersonal timing is crucial for predicting the action of the second agent, then visual detection of agent B in communicative trials should be best when the action of agent A is displayed according to the original timing (‘+0’). As delaying the onset of A's action introduces a temporal error in the prediction of B's action, performance should deteriorate with increasing distance from original timing (‘+20’, ‘+40’, corresponding to a delay of 667 ms and 1333 ms, respectively). In contrast, no effect of the timing manipulation should be expected in individual trials, as the actions of agent A and B then are unrelated.

## Methods

### Participants

Twenty undergraduate and graduate students from the University of Turin (8 male and 12 female, mean age  = 24 years, age range 18–31) volunteered to take part in the experiment. All had normal or corrected-to-normal vision, had provided informed consent, and were naïve with respect to the purpose of the study. The experiment was conducted in accordance with the ethical standards laid down in the 1964 Declaration of Helsinki.

### Stimuli

Stimuli consisted of two point-light figures with 13 markers indicating the head and the center of the major joints of each actor (shoulders, elbows, wrists, hips, knees, and feet). Two action sequences were selected from the Communicative Interaction Database [21]: a communicative action sequence and an individual action sequence. The *communicative sequence* displayed a communicative interaction between two agents (A and B): Agent A asks agent B to squat down, and agent B squats down. The *individual sequence* was created by substituting agent A's communicative action with a non-communicative action with the same onset and duration: Agent A turns around, and agent B squats down. Stimuli were constructed in accordance with the motion capture procedures described in detail in Dekeyser, Verfaillie, and Vanrie [22]. For the communicative sequence, the actions of the two actors were captured at the same time, in order to guarantee that B's response matched A's communicative gesture in all respects (e.g., timing, position, and kinematics). The distance between A and B during stimulus acquisition was about two meters. For the individual sequence, A's action was captured while the actor was acting alone, and was then coupled with B's action. The duration of each action sequence was 3600 ms.

**Figure 1:**
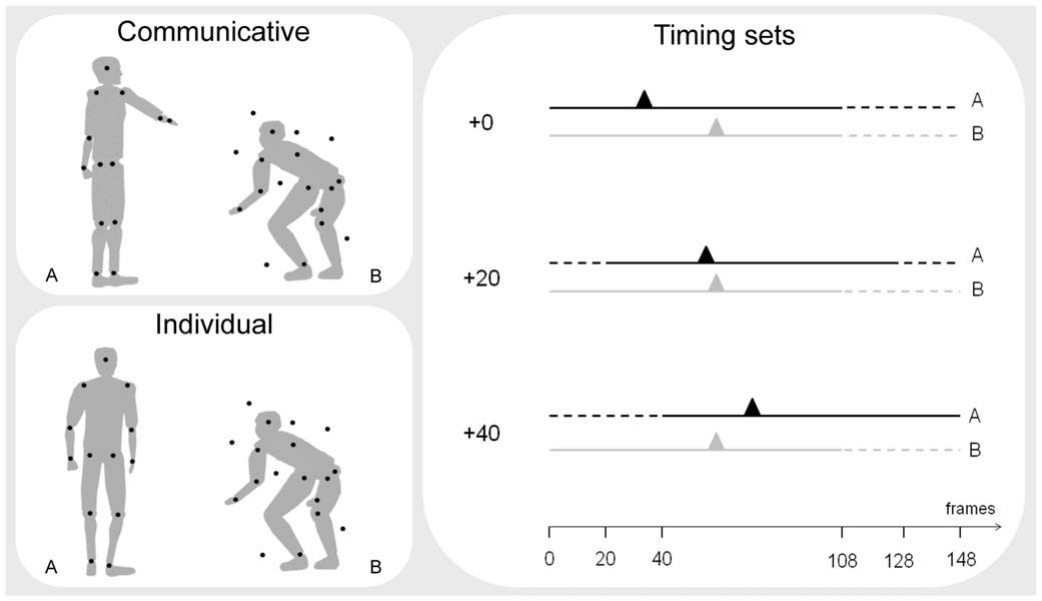
Experimental conditions. *Left, upper panel*. Example of communicative signal trial (single frame). Agent A asks agent B to squat down; agent B squats down. B is presented using limited-lifetime technique and masked with temporally scrambled noise dots. *Left, lower panel*. Example of individual signal trial (single frame). Agent A turns around; agent B squats down. *Right panel*. Representation of the three timing sets (‘+0’, ‘+20’ and ‘+40’). The duration of the action of agent A (black line) and B (gray line) in both the communicative and the individual condition is 148 frames (4933 ms). Triangles indicate key kinematic landmarks characterizing the observed action sequences. The black triangles represent the moment in time in which A's hand begins to move up to signal B the up/down movement path (first change in the vertical position compared to frame 0 of the right wrist dot). The gray triangles represent the moment when B starts to squat down (first change in the vertical position compared to frame 0 of the right hip dot). Dashed lines represent static frames added to equate stimulus duration across timing sets.

For each type of action sequence (communicative versus individual), three different temporal sets were assembled: ‘+0’, ‘+20’, ‘+40’ frames. The ‘+0’ motion set was assembled to reproduce the original timing of the actions of the two agents. In the ‘+20’ motion set, 20 static frames were added at the beginning of A's action, so that the onset of A's action was delayed by 667 ms (frame rate was 30 frames/s). In the ‘+40’ motion set, 40 static frames were added at the beginning of A's action, so that the onset of A's action was delayed by 1333 ms. To equate stimulus duration, 40 static frames were added at the end of agent B's action in the three temporal sets. The resulting duration for each stimulus was 148 frames (corresponding to 4933 ms; see Figure 1).

#### Stimuli identification

Before testing the impact of the timing manipulation on the detection of biological motion in a mask, we performed a preliminary experiment aimed at evaluating the effects of the timing manipulation on stimulus identification (in the absence of a mask). 111 students from the Faculty of Psychology at the University of Turin (12 male and 100 female; mean age  = 22.1 years, age range  = 19–58) took part in the experiment. All participants were naive as to the purpose of the study and had no previous experience with point-light displays. None of the participants involved in the preliminary experiment took part in the main experiment. Participants were tested in a group setting, in a conference room with a central projection screen. Viewing distance ranged from 5 to 20 m. Participants were presented with three-quarter views (i.e., agents shown in a depth-orientation between the frontal orientation – in which the actors are facing the observer – and a sagittal orientation – in which the actors' facing direction is orthogonal to the observer's line of sight; the three-quarter view corresponded to the 125° reference orientation used in the CID [21]) of the communicative and the individual action sequences in the three timing sets (+0, +20, +40). After each stimulus presentation, they were asked to report whether the two agents were communicating or acting independently of each other. All participants (100%) correctly identified the individual action sequence as non-communicative. The communicative action sequence was correctly identified as communicative by 110 out of 111 participants (99%) in the ‘+0’ motion set. Forty-eight participants (43%) classified the communicative sequence as communicative in the ‘+20’ set. Only 8 participants (7%) classified the communicative sequence as communicative in the ‘+40’ set, suggesting that the action of agent A occurred too late for B's action to be interpreted as matching A's communicative request.

### Apparatus and procedure

Stimuli were displayed on a 15.4-inch WXGA screen (display resolution: 1280×800; refresh rate: 60 Hz) using MatLab (7.1 version) software. Viewing distance was 60 cm. Dots (subtending approximately 0.14 deg each) were black against a grey background. The visual angle between the points attached to the head and the feet was about 7.15 deg. Participants were tested individually in a dimly lit room.

A two-alternative forced-choice (2AFC) paradigm was employed. Each trial consisted of two intervals, a *target* interval (containing agent B) and a *nontarget* interval (not containing agent B), with a 500 ms fixation cross (black against a grey background) in between. In the target interval, B's actions were displayed using the limited lifetime technique (see below) and masked with limited lifetime noise dots [13], [23] (see Figure 1). Each signal dot was presented for a fixed duration or ‘lifetime’ (200 ms) at one of the 13 possible locations, then disappeared, and reappeared at another randomly chosen location. Six signal dots per frame were shown. However, dot appearance and disappearance were asynchronous across dots in order to avoid motion transients from simultaneous transitions of all sampling dots. Thus, the 200 ms lifetime of a single dot partly overlapped with the 200 ms lifetime of other dots. Note that this implies that the visible lifetime of some dots was actually shorter in the first 200 ms and last 200 ms of the stimulus presentation. For these dots lifetime already started or should proceed but was partly not visible because the presentation of the point-light stimulus had not yet started or had already ended, respectively. Noise dots had the same trajectories, size, and duration as the signal dots, but were temporally and spatially scrambled (they appeared in an area subtending approximately a 8.6°×14.3° region and were displayed for the whole duration of the stimulus, i.e., 148 frames; see Stimuli section). The number of noise dots was adjusted individually for each participant during a training session (see below).

In the nontarget interval, agent B was substituted by limited lifetime scrambled dots obtained by temporally scrambling the signal action. Noise dots were also added so as to obtain the same number of dots as displayed in the target interval. On average, positions and motions of the dots in the nontarget interval equaled those of the target interval (see also [13]). In both the target and the nontarget intervals, A was neither limited lifetime nor masked.

Participants were asked to decide which interval contained agent B as opposed to no agent. Responses were given by pressing one of two keys on a keyboard (a left key when the target interval was presented as the first interval, a right key when the target interval was presented as the second interval). To prevent participants from using the onset of B's action as a cue to accomplish the task, +10, 0, or −10 static frames were randomly added at the beginning (and at the end) of all action sequences. Each participant completed three blocks of 36 trials (2 types of action sequence, by 3 timing sets, by 18 repetitions). Each block consisted of trials of communicative and individual action sequences in the three timing sets presented in a randomized order. Blocks lasted approximately seven minutes each and were separated by a rest period of one minute. Accuracy feedback was given after each block. Before starting the experiment, participants completed a training session, in which they were presented with the actions of a single agent masked with different levels of noise dots (see below).

### Training session

Before the actual experiment, the number of noise dots was adjusted individually for each participant during a training session. Each trial consisted of two intervals, a *target* interval (containing agent B) and a *nontarget* interval (not containing agent B), with a 500 ms fixation cross in between. In the target interval, B's ‘squatting down’ action (148 frames) was displayed using the limited lifetime technique and masked with limited lifetime noise dots (see Apparatus and Procedure section). In the nontarget interval, agent B was substituted by limited lifetime scrambled dots obtained by temporally scrambling the signal action. Noise dots were also added so as to obtain the same number of dots as displayed in the target interval. Participants were presented with five levels of noise (5, 10, 20, 40, and 80 noise dots) in a 2AFC task. Each participant completed three blocks of 30 trials (5 noise levels by 18 repetitions). Trials in each block were presented in a randomized order. Individual noise levels were determined by fitting a cumulative Gaussian function to the proportion of correct responses and determining the 75% threshold. The minimum noise level allowed was five noise dots (M = 28.3, SD = 26.4).

## Results

The mean proportion of correct responses was .78 (score range = .62–.93). In order to compare participants' performance for communicative and individual action sequences in the three timing sets, criterion (*c*) and sensitivity (*d*') parameters were extracted [24]. For each participant we calculated the proportion of *hits* (arbitrarily defined as “first interval” responses when the target was in the first interval) and *false alarms* (“first interval” responses when the target was in the second interval) for each type of sequence in the three timing sets. Proportions of 0 were replaced with 0.5/N, and proportions of 1 were replaced with (N-0.5)/N (where N is the number of “first interval” and “second interval” trials).

Criterion values ranged from −.40 to .54 (*M* = .04, *SD* = .28) for the communicative action sequence (+0, *M* = .06, *SD* = .33; +20, *M* = −.02, *SD* = .28; +40, *M* = .08, *SD* = .53), and from −.40 to .43 (*M* = −.04, *SD* = .21) for the individual action sequence (+0, *M* = .02, *SD* = .29; +20, *M* = −.06, *SD* = .42; +40, *M* = −.08, *SD* = .25). In none of the experimental conditions did *c* differ from zero (one-sample t-test, *t* ranging from −1.35 to .88; *p* ranging from .192 to .752), indicating that participants' responses were unbiased (i.e., there was no systematic tendency to respond ‘first interval’ or ‘second interval’). An analysis of variance (ANOVA) with type of action sequence (communicative vs. individual) and timing set (+0, +20, +40) as within-subjects factors revealed no significant effect.

**Figure 2:**
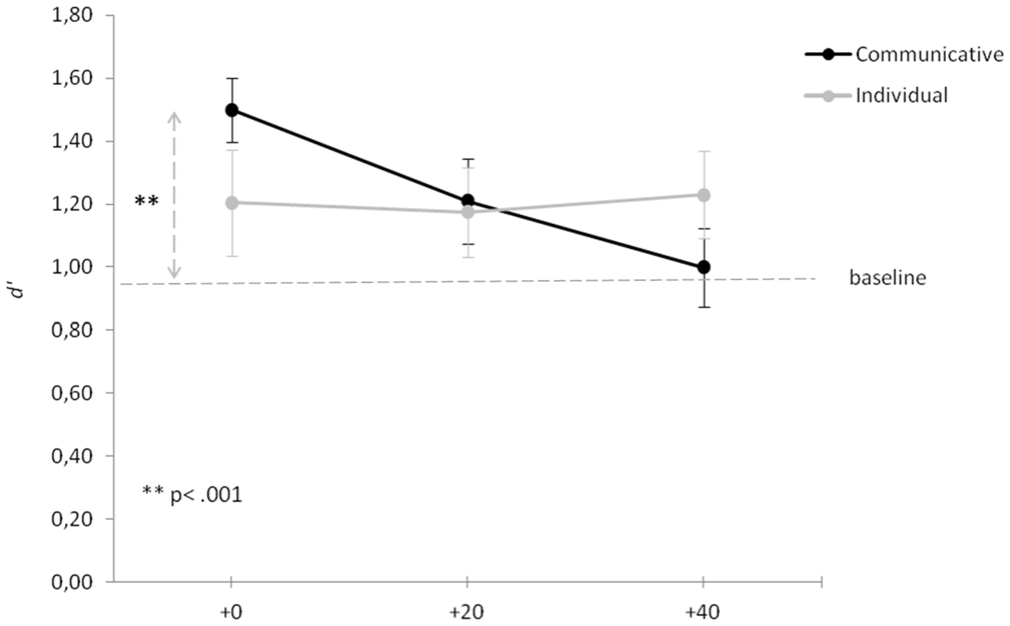
Sensitivity. Sensitivity (*d*') for the communicative (black line) and individual (gray line) condition in the three timing sets. Error bars represents standard errors. The dashed line (*d*' = .95) represents the 75% correct responses level selected during the training session, in which agent B was presented alone.

Sensitivity values ranged from .46 to 1.97 (*M* = 1.24; *SD* = .41) for the communicative action sequence (+0, *M* = 1.50, *SD* = .46; +20, *M* = 1.21, *SD* = .60; +40, *M* = 1.00, *SD* = .56) and from .07 to 2.07 (*M* = 1.20; *SD* = .54) for the individual action sequence (+0, *M* = 1.20, *SD* = .76; +20, *M* = 1.17, *SD* = .64; +40, *M* = 1.23, *SD* = .62) (see Figure 2). An ANOVA on *d*' with type of action sequence (communicative vs. individual) and timing set (+0, +20, +40) as within-subjects factors revealed a significant interaction effect between type of action sequence and timing set (*F*_(2,38)_ = 3.38; *p* = .045). No main effect of type of action sequence (*F*_(1,19)_ = .08; *p* = .776) and timing set (*F*_(2,18)_ = 2.60; *p* = .087) was found.

In order to further explore the interaction between type of action sequence and timing set, *d*' for the communicative and the individual action sequences were submitted to separate ANOVAs, with timing set as within-subjects factor. For the communicative action sequence, results revealed a significant effect of timing set (*F*_(2,38)_ = 6.65; *p* = .003), with *d*' decreasing linearly from the ‘+0’ motion set, to the ‘+20’ motion set, to the ‘+40’ motion set (linear contrast, *F*_(1,19)_ = 19.00; *p*<.001). For the Individual condition, no significant effect of timing set was found (*F*_(2,38)_ = .06; *p* = .940).

### Enhancement by congruent pairing versus disruption by incongruent pairing

The main effect of timing set on *d*' for the communicative action sequence may have arisen in one of two ways: enhancement by congruent pairing with the action of agent A, or disruption by incongruent pairing with the action of agent A (or both). To distinguish between these two possibilities, we compared *d*' data points from the main experiment with those obtained in the training session, where the action of agent B was displayed in isolation. The noise level selected at the end of the training session corresponded to 75% of correct responses, i.e., *d*' = .95 (assuming *c* = 0; see dashed line in Figure 2). In the main experiment mean *d*' (across action sequences and timing sets) was significantly greater than .95 (*t*_(19)_ = 2.94; *p* = .009), suggesting a general enhancement in performance (likely due to a practice effect). For the communicative action sequence, enhancement by congruent pairing was evident in the ‘+0’ motion set (*t*_(19)_ = 5.34; *p*<.001). By contrast, *d*' did not significantly differ from .95 in the ‘+20’’ set (*t*_(19)_ = 1.92; *p* = .070) and in the ‘+40’’ set (*t*_(19)_ = .38; *p* = .708). For the individual action sequence, no enhancement or disruption was found at any time shift (*t* ranging from 1.51 to 2.01; *p* ranging from .059 to .148). This suggests that visual discrimination of agent B in communicative trials was enhanced by congruent pairing with the action of agent A, rather than disrupted by incongruent pairing.

### Correlation between *d*' and action identification

To explore the role of interpersonal predictive coding further, we verified whether visual detection of agent B in communicative trials correlated with the impact of the timing manipulation on recognisability of the stimuli. To test this, we correlated the percentage of participants who correctly identified the communicative action sequence as communicative in the three timing sets (measured independently in a preliminary experiment, see Methods section, Stimulus identification) with *d*' in the three timing sets. Stimulus identification was found to be positively correlated with *d*' (*r*_(1)_ = 1.00; *p* = .022). This suggests that the more the action sequence was identifiable as communicative, the better observers were able to detect the presence of agent B.

## Discussion

To successfully engage in interactive activities such as carrying a table together, dancing a waltz, or playing football, actors must be able to adjust their actions to those of the other person, choosing an appropriate complementary action to be performed at an appropriate time. This requires the ability to predict *what* others will do next, but also *when* their actions will take place, i.e., to generate predictions about the timing of others' actions [20].

Previous evidence suggests that, in the context of interactive activities between two agents, the actions of one agent can be used to predict what the second agent will do, even if no physical contingency between the actions of the two agents is implied [14], [15]. Here we show for the first time that interpersonal predictive coding incorporates timing aspects: Temporal coupling between the actions of two agents (A and B) engaged in a communicative interaction increased the participants' ability to detect the presence of the second agent (agent B). Detection performance deteriorated linearly as the action of agent A was delayed with respect to the original timing. In contrast, no effect of the timing manipulation was observed for individual trials, in which the action of agent A was not related to the action of agent B. These findings suggest that, in the context of communicative interactions, the actions of one agent are used to generate predictions about the *timing* of the action of the second agent. The question arises how this can be achieved.

According to current theories of motor control, observers generate precisely timed predictions about the sensory consequences of their actions whenever they plan to move, relying on so-called forward models [25]. It has been proposed that these already existing real-time forward models for the control of one's own actions may also be used to predict the actions of others [17], [18], [26]–[31]. Observing others' actions activates corresponding representations in the observer's motor system, and these representations might enable the observer to generate predictions by running real-time internal simulations. In this account, perceptual and motor systems share representations for actions [32] and the same predictive mechanism used to anticipate the sensory consequences of one's own movement may be employed to predict the timing of others' actions.

Findings on real-time simulation processes support this idea [17], [18], [33], [34]. Graf and colleagues [17] demonstrated that internal action simulation runs time-locked to the real action, even when the action is covered by an occluder. Observers viewed brief videos of point-light actions, followed by an occluder and a static test posture. They were instructed to judge whether the test stimulus depicted a continuation of the action in the same orientation or in a different orientation. Results showed that performance was best when occluder time and movement gap corresponded, and decreased with increasing time distance. Adding a concurrent motor task, Springer and colleagues [34] found that this distance function is modulated by motor execution. This supports the assumption that i) action prediction operates in real-time; and ii) motor processes are involved in real-time predictions.

One way in which such real-time simulation may contribute to interpersonal predictive coding is by running multiple simulations [35], [36]. Observers may run multiple action simulations to predict how, at any moment, the action of one agent should relate to the action of a second agent. This way, motor simulation might support various forms of interpersonal coordination, from resonant imitation to more complex forms of joint and complementary action, where the spatiotemporal features of one individual's behavior are different from, but systematically related to, those of another individual [37].

Another, more simple, way in which the timing of the second agent's action may be predicted in the context of a communicative interaction is by shifting from symmetrical simulation to reciprocity. Using single-pulse transcranial magnetic stimulation, Sartori and colleagues [38], [39] found that observation of an action sequence does not inevitably lead to symmetrical simulation: When the action evokes a complementary response, a shift from symmetrical simulation to reciprocity is observed in the participants' corticospinal activity. This shift appears to take place at an early stage of action observation, suggesting that observers rely on advanced motor information to anticipate the agent's intention and prepare a appropriate complementary response [11], [12], [40] (for review, see [10]). Provided that the actions of two agents are related in time, the transition from symmetrical simulation to reciprocity during observation of communicative actions might be used to predict ‘what’ aspects, but also ‘when’ aspects of the second agent's response. Future studies measuring motor activation during interpersonal predictive coding may help to clarify whether and to what extent modulation of motor excitability during observation of agent A's action is predictive of B's response.

A second issue to be addressed by future investigations is how real-time predictions interact with high-level processes postulated in theory of mind and communication research such as mental state attribution. A range of behavioral and neuroscientific studies has provided evidence that attribution of intention is deeply rooted in the actions of interacting agents [41], [42]. The finding that stimulus identification correlates with real-time predictions complements previous research suggesting that observer take interpersonal timing into account when evaluating the semantics of an interaction. In the present work we only considered three delays. Studies using finer temporal resolution may help to understand how close real-time predictions and high-level processes and representations mirror interpersonal timing.
